# Nanoparticles: a promising tool against environmental stress in plants

**DOI:** 10.3389/fpls.2024.1509047

**Published:** 2025-01-27

**Authors:** Xu Zhou, Ahmed H. El-Sappah, Amani Khaskhoussi, Qiulan Huang, Amr M. Atif, Mohamed A. Abd Elhamid, Muhammad Ihtisham, Mohamed F. Abo El-Maati, Salma A. Soaud, Walid Tahri

**Affiliations:** ^1^ International Faculty of Applied Technology, Yibin University, Yibin, Sichuan, China; ^2^ College of Agriculture, Forestry, and Food Engineering, Yibin University, Yibin, Sichuan, China; ^3^ Department of Genetics, Faculty of Agriculture, Zagazig University, Zagazig, Egypt; ^4^ Key Laboratory for Green and Advanced Civil Engineering Materials and Application Technology of Hunan Province, College of Civil Engineering, Hunan University, Changsha, China; ^5^ Department of Microbiology, Faculty of Agriculture, Zagazig University, Zagazig, Egypt; ^6^ Agriculture Biochemistry Department, Faculty of Agriculture, Zagazig University, Zagazig, Egypt

**Keywords:** abiotic stress, biotic stress, secondary metabolic response, molecular response, and nanotechnology

## Abstract

With a focus on plant tolerance to environmental challenges, nanotechnology has emerged as a potent instrument for assisting crops and boosting agricultural production in the face of a growing worldwide population. Nanoparticles (NPs) and plant systems may interact molecularly to change stress response, growth, and development. NPs may feed nutrients to plants, prevent plant diseases and pathogens, and detect and monitor trace components in soil by absorbing their signals. More excellent knowledge of the processes of NPs that help plants survive various stressors would aid in creating more long-term strategies to combat these challenges. Despite the many studies on NPs’ use in agriculture, we reviewed the various types of NPs and their anticipated molecular and metabolic effects upon entering plant cells. In addition, we discussed different applications of NPs against all environmental stresses. Lastly, we introduced agricultural NPs’ risks, difficulties, and prospects.

## 1 Introduction

Plants are exposed to a range of environmental stressors that have the potential to diminish and restrict the yield of crops (El-Sappah and Rather, 2022; Ihtisham et al., 2023). Plants encounter two distinct categories of environmental challenges: abiotic stress and biotic stress (El-Sappah and Rather, 2022). The global loss of essential crop plants is attributed to abiotic stressors such as radiation, salinity, floods, drought, temperature extremes, heavy metals (HMs), and other factors (Umar et al., 2021). Conversely, biological stressors encompass the presence of pathogens such as fungi, bacteria, oomycetes, nematodes, and herbivores (Bhatla et al., 2018). Crop production losses can be attributed to biotic and abiotic stressors (Gull et al., 2019). The overproduction of reactive oxygen species (ROS) is identified as a significant factor contributing to crop losses resulting from abiotic stressors (El-Sappah et al., 2022b; Li et al., 2023b). In recent decades, significant endeavors have been undertaken to enhance agricultural productivity by extensively employing chemicals that have enduring and profound impacts on the environment and human wellbeing. Consequently, innovative technology is necessary to nourish the global population while minimizing environmental damage (Gill and Garg, 2014; Tudi et al., 2021).

The field of nanotechnology is stimulating and undergoing significant advancements, leading to many breakthroughs (Ratner and Ratner, 2003; Abbas et al., 2022b; Naveed et al., 2022b). Nanotechnology has the potential to offer viable solutions to agricultural challenges and contribute to the attainment of a sustainable and secure future for the agricultural sector (Pandey, 2018). In recent years, nanotechnology has garnered significant attention due to its wide range of applications in medical, drug delivery, energy, poultry production, and agri-food industries (Ashraf et al., 2021). According to Khan and Upadhyaya (2019), nanoparticles (NPs) are diminutive substances with dimensions spanning from 1 to 100 nm. Unlike larger-sized entities, NPs possess unique and varied physicochemical characteristics (Hsiao and Huang, 2011). The high surface area-to-volume ratio, high adsorption efficacy, and higher connecting and working efficiencies of NPs can be attributed to their small size (Gil et al., 2010). NPs may help plants resist stress by improving nutrient uptake by assisting plants in absorbing and transporting minerals and nutrients (Singh et al., 2024), improving photosynthetic efficiency (Faizan et al., 2021), regulating hormone balance, particularly auxins and gibberellins, scavenging ROS (Tripathi et al., 2022), which can harm plants, and improving stress-responsive gene expression by boosting the expression of genes that help plants react (Abideen et al., 2022).

The current review comprehensively summarizes the advances in applying nanobiotechnology in agriculture, especially the potential of biosynthesized NPs to relieve abiotic and biotic problems in crop production.

## 2 Properties of nanoparticles

NPs have been classified into four categories: electrical and optical, magnetic, mechanical, and thermal (Khan et al., 2019), as shown in **Table 1**.

**Table 1:** Overview of nanoparticle properties, measurement methods, and agricultural applications.

Property	Measurement method	Typical agricultural applications	Example nanomaterials	References
**Electrical**	Electrical conductivity and impedance spectroscopy.	Improving soil conductivity and using electrochemical sensors to monitor moisture and nutrients.	Carbon nanotubes with graphene oxide.	(Kashyap and Kumar, 2021; Siddiqui and Aslam, 2023)
**Optical**	UV–visible and fluorescence spectroscopy.	Light-activated pesticide delivery and photothermal treatment for pathogen control.	Gold nanoparticles (Au-NPs) and quantum dots.	(Cabral and Baptista, 2014; Li et al., 2024)
**Magnetic**	Magnetic susceptibility and hysteresis loop.	Soil remediation and targeted use of agricultural pesticides.	Iron oxide (Fe_3_O_4_) and magnetite NPs.	(Zhou et al., 2016; Le Wee et al., 2022)
**Mechanical**	Nanoindentation and atomic force microscopy.	Improving plant cell wall strength, resilience to physical stress, and seed germination.	The NPs made of silica (SiO_2_) and chitosan.	(Husen and Jawaid, 2020; Nour, 2024)
**Thermal**	Differential scanning calorimetry and thermal conductivity measurements.	Heat stress reduction, temperature modulation in plants, and improved thermal stability of fertilizers.	Titanium dioxide (TiO_2_) with copper nanoparticles (Cu-NPs).	(To et al., 2020; Chettri et al., 2024)

Note: The electrical property of nanoparticles is about how well they can let electricity pass through or block it. The optical property of nanoparticles involves their interaction with light, such as absorption, scattering, and fluorescence, enabling applications like light-activated pesticide delivery and pathogen control. The magnetic property of nanoparticles enables manipulation via magnetic fields for soil remediation and pesticide targeting. The mechanical property of nanoparticles enhances strength, elasticity, and resilience in plant tissues and seed. The thermal property of nanoparticles regulates.

NPs have a higher degree of interdependence between their electrical and optical properties. NPs composed of noble metals exhibit optical properties that depend on their size and possess a distinct ultraviolet–visible (UV–Vis) extinction band absent in the bulk metal spectrum (Gautam et al., 2021). The phenomenon known as localized surface plasma resonance (LSPR) arises when the frequency of incident photons remains constant while the conduction electrons are collectively excited (Singh, 2017). The LSPR excitation induces absorption at specific wavelengths due to resonance with a significantly high molar excitation coefficient (Kochuveedu and Kim, 2014). LSPR improves NPs’ capacity to interact with light, which may be employed realistically to enhance plant health, disease management, and precision agricultural approaches.

The magnetic properties of these NPs have garnered significant attention from researchers in various domains, including heterogeneous and homogeneous catalysis, biomedicine, magnetic fluids, data storage, magnetic resonance imaging, and environmental remediation, such as water decontamination (Ali et al., 2021). According to existing scholarly works, NPs exhibit optimal performance within the critical size range of 10–20 nm (Reiss and Hütten, 2005). The magnetic characteristics of NPs were more dominant at a tiny scale, rendering these particles highly desirable and valuable in a wide range of applications (Cardoso et al., 2018). NPs exhibit magnetic properties due to their non-uniform electronic dispersion (Karunakaran et al., 2018). The qualities mentioned are influenced by the synthetic process, which can be achieved in many ways, such as solvothermal (Navas et al., 2020), co-precipitation, micro-emulsion, thermal breakdown, and flame spray synthesis (Dippong et al., 2021).

Conversely, NPs’ unique mechanical characteristics allow researchers to explore innovative applications in diverse critical domains, including tribology, surface engineering, nanofabrication, and nanomanufacturing (Khan et al., 2019). Diverse mechanical metrics like elastic modulus, hardness, stress and strain, adhesion, and friction can be examined to ascertain the precise mechanical characteristics of NPs (Wu et al., 2020). Various factors, including surface coating, coagulation, and lubrication, influence NPs’ mechanical properties (Balmert and Little, 2012; Pownraj and Valan Arasu, 2021). There are distinct mechanical properties between NPs and titles and their bulk materials. In a lubricated or greased contact, the stiffness contrast between the NPs and the external surface determines whether the NPs are indented into the plan surface or distorted when the pressure at contact is sufficiently high (Madkour and Madkour, 2019). These crucial data may provide insights into the performance of the NPs in a contact scenario. It is of utmost importance to exercise control over the mechanical properties of NPs and their interactions with various surface types to enhance surface quality and remove more material (Khan et al., 2019).

A comprehensive comprehension of the essential mechanical characteristics of NPs, including their elastic modulus and hardness, movement law, friction and interfacial adhesion, and size dependence, is typically required to achieve successful results in these areas (Mehmood et al., 2023). Metal NPs are widely recognized for their superior thermal conductivities compared to solid fluids. At room temperature, the thermal conductivity of copper is approximately 700 times greater than water’s and nearly 3,000 times greater than engine oil’s (Choi and Eastman, 1995). Thermal conductivity is stronger in oxides such as alumina (Al_2_O_3_) than in water (Senthilraja et al., 2015). It is anticipated that fluids containing suspended solid particles will exhibit considerably greater thermal conductivities than current heat transfer fluids (Choi and Eastman, 1995). Nanofluids are generated through the dispersion of solid particles at nanometric wavelengths within liquid mediums, such as water, ethylene glycol, or oils (Taylor et al., 2013). The properties of nanofluids are anticipated to surpass those of traditional heat transfer fluids and fluids containing particles at the tiny level (Das and Stephen, 2009). Because of heat transmission at the particle’s surface, particles with a substantial total surface area are favored. The enhancement of suspension stability is also attributed to the significant total surface area (Timofeeva et al., 2009). Another study demonstrated that nanofluids containing Al_2_O_3_ or CuO-NPs (copper oxide nanoparticles) in ethylene (H₂C=CH₂) or water had increased thermal conductivity (Mohamad et al., 2018).

## 3 Plants’ molecular and metabolic responses to nanoparticle exposure

Assessing the absorption, dispersion, and toxicity of NP exposure in plants requires understanding the nature of NP interactions with plants. According to Dietz and Herth (2011), NPs’ enormous surface area, minuscule size, and innate catalytic reactivity are the leading causes of their chemical and mechanical interactions with biological systems, such as plants.

**Figure 1:**
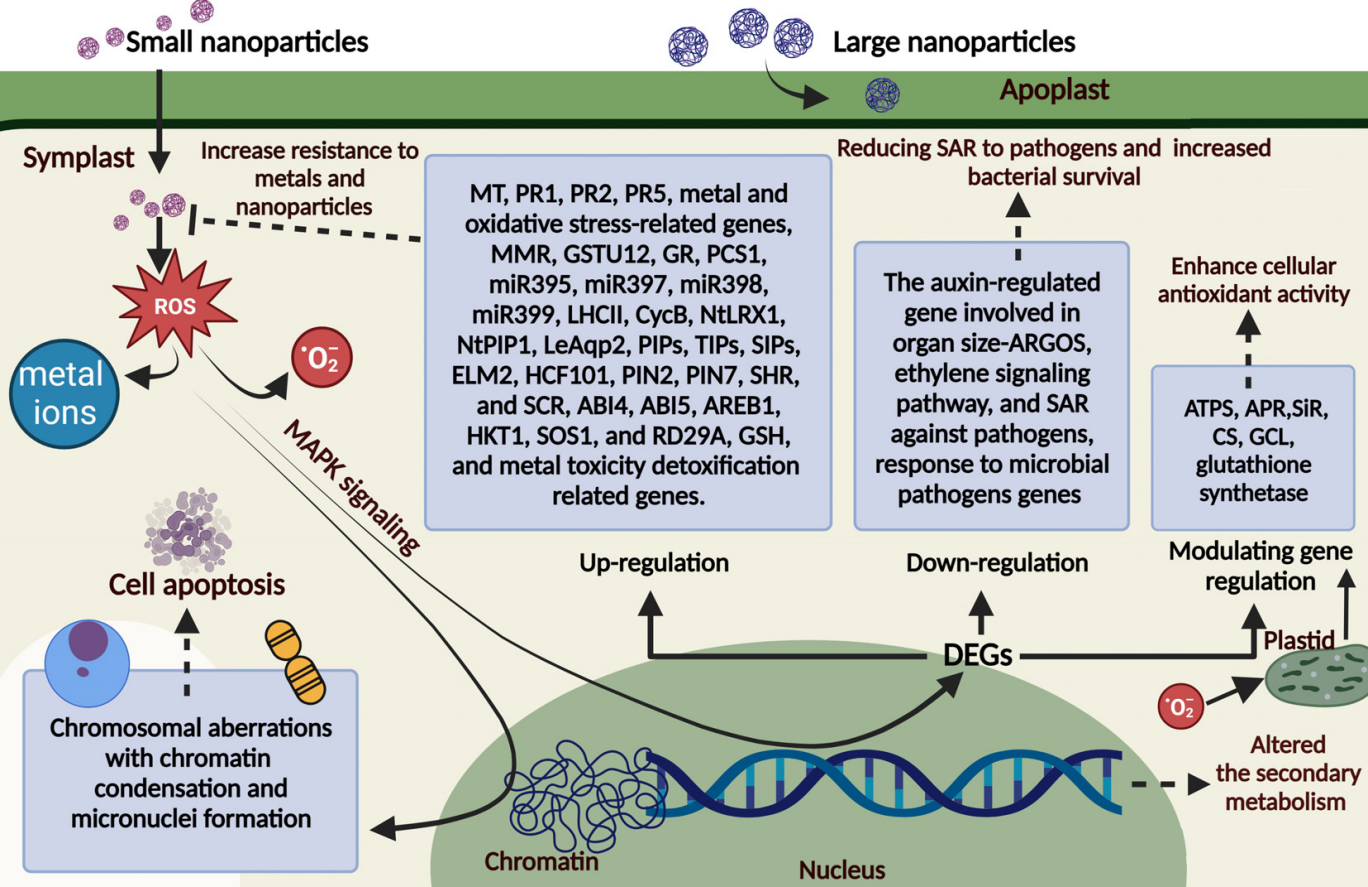
The plant cell’s molecular response to NP exposure. Small and large nanoparticles infiltrate plant cells, triggering a process termed MAPK signaling, leading to reactive oxygen species (ROS) generation. Numerous genes have been identified as overexpressed in response to NPs, including metallothionein (*MT*), pathogenesis-related genes (*PRs*: *PR1*, *PR2*, and *PR5*), DNA mismatch repair (*MMR*) genes, proliferating cell nuclear antigen (*PCNA*), glutathione S-transferase (*GSTU12*), glutathione reductase (*GR*), and phytochelatin synthase (*PCS1*); specific microRNAs (*miR395*, *miR397*, *miR398*, and *miR399*); the light-harvesting complex II (*LHCII*) b gene; and various abiotic stress-related genes, including those involved in oxidative stress, salinity, water management, sulfur metabolism, glutathione (*GSH*) biosynthesis, metal toxicity detoxification, cell division (*CycB*), cell wall extension (*NtLRX1*), water transport (aquaporin gene *NtPIP1*), aquaporin gene (*LeAqp2*), plasma membrane intrinsic proteins (*PIPs*), tonoplast intrinsic proteins (*TIPs*), small and essential intrinsic proteins (*SIPs*), and tolerance to cadmium sulfide quantum dots (MYB-containing gene *ELM2*), as well as genes implicated in high chlorophyll photosynthesis. Additional genes associated with hormonal stimuli and plant defense systems [e.g., the ethylene signaling pathway, systemic acquired resistance to pathogens, and the auxin-regulated gene linked to organ size (ARGOS)] were downregulated after NP treatment. It diminishes the expression of genes associated with microbial infection defense, enhancing bacterial colonization and survival while decreasing self-protection against pathogens. Additional genes affected by NP exposure are glutathione synthetase (GS), 3′-phosphoadenosine 5′-phosphosulfate reductase (APR), sulfite reductase (SiR), cysteine synthetase (CS), glutamate-cysteine ligase (GCL), and ATP sulfurylase (ATPS). This figure was created using BioRender.

It is commonly known that NPs alter biological architecture in various complex and little-understood ways. The effects of environmental stress on plants have been evaluated using multiple biochemical markers, including metabolite composition, membrane integrity, and enzyme activity (Singh et al., 2020). A physiological study indicates that smaller NPs, like plasmodesmata, can flow through the symplast, but larger NPs congregate in the apoplastic region (Banerjee et al., 2019; Wang et al., 2023), as seen in **Figure 1**. The most commonly reported mechanisms of NP toxicity in plants are the release of toxic metal ions (Dietz and Herth, 2011), increased production of ROS leading to oxidative stress (OS) (Asli and Neumann, 2009; Dietz and Herth, 2011), and mechanical damage or clogging of pores caused by cell surface coating (Pashkow et al., 2008).

OS is one of the most widely observed stressors caused by NP exposure at the cellular level (Horie and Tabei, 2021). It is widely described as an imbalance between antioxidant activity and oxidant production (Pisoschi and Pop, 2015). Furthermore, OS can be induced by elevated ROS levels at the expense of antioxidants. ROS, such as peroxynitrite (ONOO−), nitric oxide (NO), hydroxyl radical (∙OH), hydrogen peroxide (H_2_O_2_), and superoxide radical (O2∙−), are typically produced as by-products of biochemical reactions like neutrophil-mediated phagocytosis, enzymatic metabolism of cytochrome P450, and mitochondrial respiration (Cuzzocrea, 2006). ROS attacks nucleic acids, proteins, lipids, and most essential biomolecules, activating the NADPH-like system, impairing the electron transport chain (ETC), depolarizing the mitochondrial membrane, and damaging the mitochondrial structure (Egbuna et al., 2021). OS is a significant drawback of NP use because it can generate oxidants and promote ROS production due to the relative stability of free radical intermediates on particles’ reactive surfaces, NP-induced cellular response, or NP functionalization’s redox-active groups, which can interfere with cellular uptake (Manke et al., 2013). Such imbalances caused by NPs, whether directly or indirectly, may have serious consequences, including cytotoxicity (Saifi et al., 2018). The ROS produced by NPs may harm genetic materials, including DNA crosslinking, strand breaking, and genetic mutations (Rim et al., 2013). NPs may also boost ROS generation by stimulating inflammatory cells like neutrophils (Yang et al., 2019).

Much research has been done using models of algae, monocotyledonous plants, and dicotyledonous plants to examine the effects of various NP types on secondary metabolite precursors (Selvakesavan et al., 2023). Research on the impact of NPs on the pentose phosphate pathway, glycolysis, tricarboxylic acid cycle, and the roles played by organic acids and carbohydrates in these processes has been conducted extensively (Majumdar et al., 2019). Defense mechanisms trigger many metabolic pathways and chemicals in reaction and their supplemental roles as chelators and osmoprotectors (Singh et al., 2023). In addition, silver (Ag), copper-oxide (CuO), copper (II) hydroxide Cu(OH)_2_, cadmium oxide (CdO), cerium dioxide (CeO_2_), graphene-based, tungsten disulfide (WS_2_), and fullerols (C60) changed the fatty acid and lipid contents of *Arabidopsis thaliana*, *Cucumis sativus*, *Zea mays*, *Hordeum vulgare*, and *Phaseolus vulgaris* (Al-Khayri et al., 2023a). Amino acid metabolism provides a vital link between primary and secondary metabolites. Major precursors in the biosynthesis of these compounds include glucosinolates (e.g., methionine, leucine, isoleucine, phenylalanine, and tryptophan), phenylpropanoids [e.g., Ag-, CuO-, and Cu(OH)_2_-NPs, as well as Ag^+^ and Cu^2+^ ions), and alkaloids (e.g., arginine, lysine, ornithine, phenylalanine, proline, tryptophan, and tyrosine) (Barros and Dixon, 2020; Jan et al., 2021). Zinc oxide (ZnO), C60, graphene NPs, and tissues from *Z. mays*, *A. thaliana*, *Z. sativus*, and *T. aestivum* all encouraged the synthesis of additional amino acids (Hu and Zhou, 2014; Zhao et al., 2019; Chen et al., 2021; Li et al., 2021).

Many studies have examined how NPs affect plants’ secondary metabolism (Marslin et al., 2017). Cucumber, maize, and wheat exposed to Ag, pepper exposed to silicone dioxide (SiO_2_) or ferric oxide (Fe_2_O_3_), and Arabidopsis treated with CuO-NPs after foliar spray of Cu(OH)_2_ were all discovered to have shikimate and phenylpropanoid pathway products (Zhao et al., 2017; Soria et al., 2019; Feng et al., 2021; Kalisz et al., 2021). Cu(OH)_2_, CeO_2_, and soil application of CuO and CdO treatments decreased lettuce, spinach, cucumber, and barley phenylpropanoids (Večeřová et al., 2016; Zhao et al., 2016; Huang et al., 2019; Zhang et al., 2019b). Low CeO_2_-NPs induced metabolic reprogramming in *P. vulgaris* roots and leaves by affecting flavonoids and phenolic compounds (Majumdar et al., 2014). Gallic and benzoic acid concentrations in sativus increased, whereas hydroxycinnamic acid derivative concentrations decreased in response to carbon (C)- and CuO-NPs (Hong et al., 2016; Selvakesavan et al., 2023). When *Solanum lycopersicum* was treated with multiwalled carbon nanotubes (MWCTs), it produced fewer flavonoids and more anthocyanins (Mcgehee et al., 2017). Within the *Hypericum perforatum* cells, the production of phenylpropanoids was impacted by metal and metal oxide NPs (Kruszka et al., 2022). Ag, Au, Cu, and Pd metal NPs decreased the amounts of flavonoids and hydroxycinnamic acid derivatives in cells while increasing the accumulation of xanthone, prenylated xanthone, and benzophenone (Selvakesavan et al., 2023).

On the other hand, the treatment with CuO-NPs increased the amount of flavonoids in biomass (Selvakesavan et al., 2023). NPs have changed the metabolism of alkaloids, a family of chemicals with great biological importance as defensive metabolites (Salehi et al., 2018). (*S*)-corytuberine, laudanosine, and precursors of naphthyl isoquinoline alkaloids were found to be decreased in *P. vulgaris*, whereas demecolcine, caconine, and tropinone were found to be increased following foliar application of CeO_2_-NPs (Salehi et al., 2018). Hyoscyamine and scopolamine following ZnO-NP exposure, and taxane and tropane alkaloids after Ag- NP exposure all accumulated (Asl et al., 2019). Methocotype synthase (MWCT) in *S. lycopersicum* and graphene oxide quantum dots (GOQDs) in *Chlorella vulgaris* Beijerinck were shown to decrease the production of isoquinoline alkaloids (McGehee et al., 2017; Kang et al., 2019). Under biotic and abiotic stress conditions, *A. thaliana* produces the camalexin of indole phytoalexin (Kruszka et al., 2020). As a consequence of applying Ag-NPs, this substance was collected (Kruszka et al., 2020).

Understanding the molecular effects of nanomaterials is critical to evaluating possible routes for the effects observed in plants (Ma et al., 2015). RNA-sequencing (RNA-seq) transcriptome analysis is a solid tool to determine cellular responses compared to other omics techniques owing to the unequaled resolution of entire transcripts (Xiong et al., 2021). Transcriptome analysis revealed that *A. thaliana* treated with PVPAg-NPs had increased tryptophan metabolism, a precursor to camalexin (Zhang et al., 2019a). NPs also change the expression of genes in plants and microbes. Different kinds of NPs have different impacts on gene expression after exposure. Our defense hypotheses about plant-responsive genes that showed high expression under NP exposure come from the exposure of various plants such as Arabidopsis, tobacco, barley, maize, and soybean to different NPs such as silver NPs (Ag-NPs), titanium dioxide NPs (TiO_2_-NPs), zinc oxide NPs (ZnO-NPs), carbon nanotubes (CNTs), graphene oxide (GO), aluminum oxide NPs (Al_2_O_3_), cerium oxide (CeO_2_), and indium oxide (In_2_O_3_) NPs (Zhang et al., 2006; Dubos et al., 2010; Ze et al., 2011; Burklew et al., 2012; Chu et al., 2012; Dimkpa et al., 2012; Khodakovskaya et al., 2012; Landa et al., 2012; Kaveh et al., 2013; Yruela, 2013; Frazier et al., 2014; Gopalakrishnan Nair and Chung, 2014; Marmiroli et al., 2014; Nair and Chung, 2014; Wang et al., 2014; Chen et al., 2015; García-Sánchez et al., 2015; Thiruvengadam et al., 2015).

To summarize, NPs cause chromosomal anomalies such as chromatin condensation and micronuclei formation, disruption of cell division, and DNA damage, all of which lead to programmed cell death (PCD)/apoptosis (Shen et al., 2010; Panda et al., 2011; Thiruvengadam et al., 2015). They also damage chromosomes and the cell cycle (Sudhakar et al., 2001).

## 4 Application of nanoparticles in plant biotic resistance

**Figure 2:**
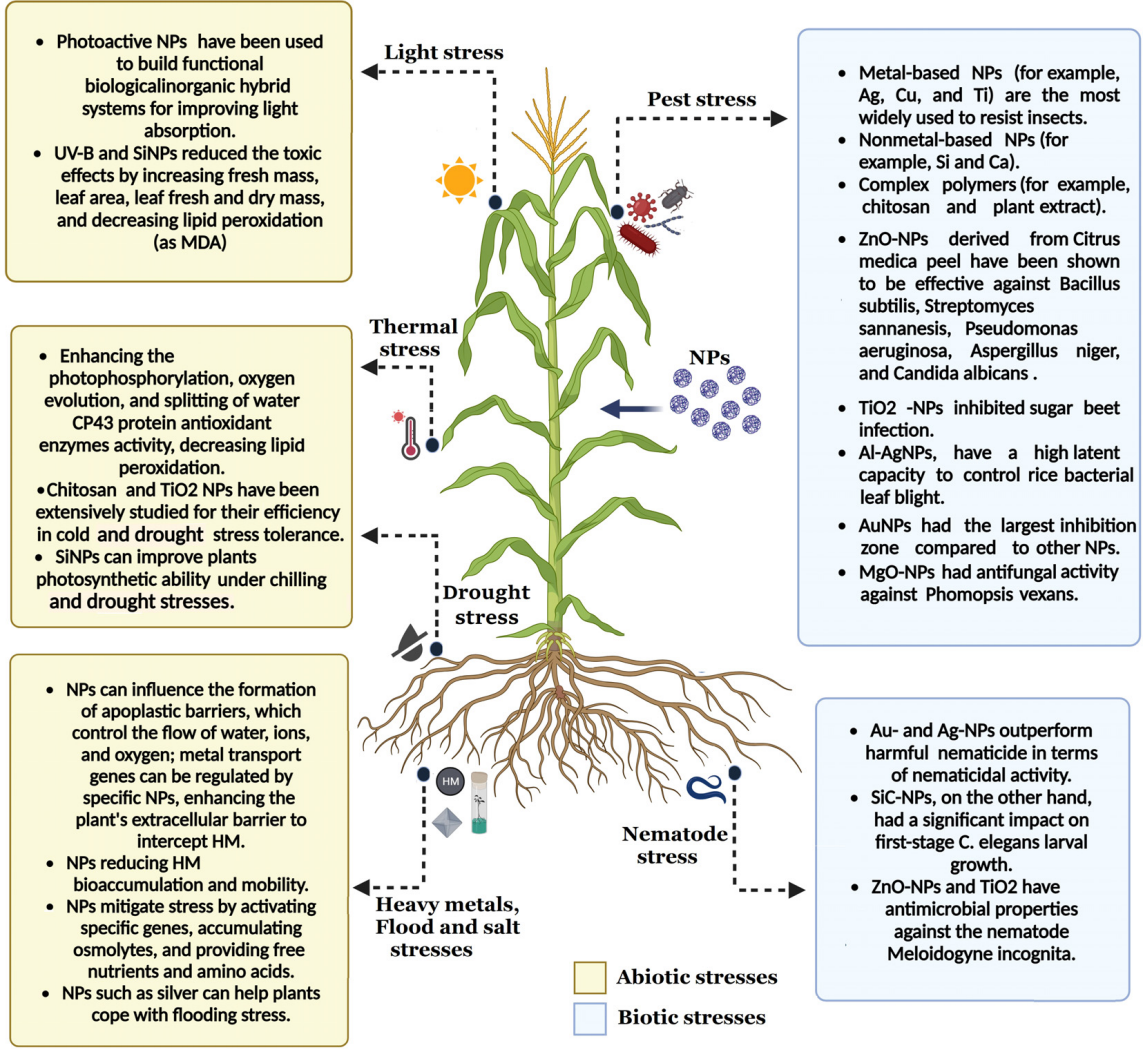
The key functions of nanoparticles in reducing the impact of different environmental stressors. Distinct effects of NPs on plant resistance have been shown, with blue representing biotic stress and yellow representing abiotic stress. This figure has been made using BioRender.

Plant health and disease control have benefited from developing several nanotechnology applications. For plant protection, multiplexed bioassays are one of the biological and non-biological uses for NPs. Hazarika et al. (2022) state that managing diseases in agriculture necessitates the employment of nano-pesticides, nano-bactericides, nano-fungicides, and nano-insecticides, along with their carefully monitored distribution. It also covers using nanosensors, nanobarcodes, and nanotubes for diagnostic purposes. Antimicrobial biomolecules and NPs can potentially eliminate harmful microorganisms, including viruses, bacteria, fungi, and yeast, in many settings, as shown in **Figure 2** (Abdelgawwad et al., 2020; Munir et al., 2023).

### 4.1 Insect infections

Insect pests represent substantial hazards to agricultural productivity, and the increased use of chemical pesticides has prompted concerns about environmental and health consequences (Nie et al., 2023). NPs have emerged as a promising option for pest control in agriculture because of their unique features, including large surface area, tiny size, and improved reactivity (Mittal et al., 2020). NPs may harm insects in various ways, causing them to perish or be unable to operate (Hazafa et al., 2021). Metal NPs, such as Ag-NPs and TiO₂-NPs, may kill insects by damaging cell structures, causing stress due to excess oxygen, and disrupting average body functioning (Saranya et al., 2020). Ag-NPs effectively control aphids (*Aphis gossypii*), whereas TiO₂-NPs and Ag-NPs significantly affect *Spodoptera litura* larvae (Jafir et al., 2021). NPs are more harmful than regular insecticides due to their small size and massive surface area, which allows them to penetrate deeper into insect bodies (Jafir et al., 2023). Carbon-based NPs, such as CNTs and polymer-based NPs, have shown promise for pest management (Yadav and Yadav, 2018). One advantage is that they may degrade organically, which benefits the environment. These NPs are generally designed to target specific pests, causing minimal damage to other organisms (Athanassiou et al., 2018).

Although NPs offer many benefits in controlling pests, there are still challenges to overcome, such as making them work better, using them more innovatively, and understanding how they affect the environment over time. More research is needed to fully realize the potential of NPs in pest control, ensuring they provide effective, sustainable, and environmentally friendly solutions for farming.

### 4.2 Fungus infection

Over 19,000 distinct types of fungi have been connected to plant diseases in agriculture worldwide (Jain et al., 2019). On the tissues of living and dead plants, they may lay dormant but alive until the conditions are right for their multiplication (Van Alfen, 2014). Certain fungi may multiply in the tissues of their hosts (Garrett, 1950). Fungus spores may be easily dispersed by soil, water, wind, and other invertebrates, including insects (Malloch and Blackwell, 1992). They might contaminate a whole crop in this way (Payne, 1998). Conversely, certain fungi benefit the host plant and could even help it grow (Mohammadi et al., 2011). One example of a mutualistic relationship is between mycorrhizae’s root systems and host plants (Johnson et al., 1997). Numerous plant diseases, including damping-off, root rot, mildew, dieback, coiled, scab, gall, blight, leaf spot, rust, and wilt, can be caused by pathogenic fungi (Jain et al., 2019). Nanotechnology is one of several methods used to mitigate the adverse effects of fungi infection in plants (Kutawa et al., 2021).

Metal NPs can be used in plant cultivation as fungicides or growth promoters (Hoang et al., 2022). The effects of Ag- and Cu-NPs on powdery mildew-affected leaves as well as on spontaneous ectomycorrhizal colonization in *Quercus robur* seedlings were documented by Olchowik et al. (2017). A significant decrease in the expansion of mycelial development was seen in spores treated with Ag-NPs (Li et al., 2022). As agricultural NPs are less harmful to people and animals, they are used far more often in plant disease control than store-bought fungicides (Malandrakis et al., 2019). Moreover, proteins, DNA, lipids, and other macromolecules can be harmed by the highly reactive hydroxyl radicals that Cu-containing fungicides can create (Demidchik, 2015). Banik and Pérez-de-Luque (2017) employed Cu-NPs to treat several plant diseases caused by *Rhizobium* spp., *Oomycetes*, *Trichoderma harzianum*, bacteria, and fungus. *Alternaria alternata*, *Phytophthora syringae*, and *P. cinnamomi* have all been found to grow less when Cu-NPs are mixed with non-nano Cu-like copper oxychloride (COC) (Munir et al., 2023). Cu-NPs may be advantageous to the agroecosystem as it has been demonstrated that they do not affect *Rhizobium* spp. or *T. harzianum*. In agricultural and food applications, ZnO-NPs can also be utilized as bactericides and fungicides (Banik and Pérez-de-Luque, 2017). ROS were produced by ZnO-NPs, damaging plant cells and triggering the plant’s defense system while improving plant growth and development (Sharma et al., 2019).

Regarding microbicidal activity, these NPs performed better than zinc particles in bulk (Siddiqi et al., 2018). Because they were tiny and had a high surface-to-volume ratio, they had good interaction with microorganisms (Ingle et al., 2014). Khan et al. (2021a) established the antibacterial and antifungal properties of Ag-NPs by utilizing them against *Fusarium avenaceum*, *Fusarium graminearum*, *Fusarium coloratum*, *Erwinia* sp., and *Pseudomonas aeruginosa*. Abdelmalek and Salaheldin (2016) assert that Ag-NPs have fungicidal action against *A. alternata*, *Alternaria citri*, and *Penicillium digitatum*. Ag-NPs are thought to possess antifungal properties against *Rhizoctonia solani*, *Botrytis cinerea*, *Macrophomina phaseolina*, *A. alternata*, *S. sclerotiorum*, and *Curvularia lunata* (Mansoor et al., 2021).

Additionally, Ag-NPs have antifungal properties against *Bipolaris sorokiniana* and *Magnaporthe grisea* (Rajeshkumar, 2019). Divya et al. (2017) report that chitosan-NPs have fungicidal efficacy against *A. alternata*, *Macrophomia phaseolina*, and *Streptococcus pneumoniae*. In addition, chitosan-NPs can be used as a fungicidal agent against *Aspergillus niger* and *F. solani* (Al-Sheikh and Yehia, 2016). On the other hand, several studies that produced Au-NPs also documented their effectiveness against various plant diseases as antifungals (Osonga et al., 2020).

Furthermore, it has been demonstrated that CuO-NPs have antifungal action against the following: *Magnaporthe oryzae, P. digitatum*, *Sclerotium rolfsii*, *R. solani*, *Colletotrichum musae*, and *B. cinerea* (Huang et al., 2015). CuO- and Cu_2_O-NPs have a fungicidal impact on *Phytophthora infestans*, as Giannousi et al. (2013) show. Evidence shows that a broad spectrum of fungi can be affected by the antifungal characteristics of different metal oxide NPs, such as Si-NPs, MgO-NPs, ZnO-NPs, and TiO_2_-NPs (Slavin and Bach, 2022). Sharma et al. (2016) provided evidence of MgO-NPs’ antifungal efficacy against *Phomopsis vexans*. Derbalah et al. (2018) state that *Alternaria solani* can benefit from silica NPs’ antifungal properties.

Additionally, Park et al. (2006) reported that *M. grisea*, *R. solani*, *Pseudomonas syringae*, *Xanthomonas compestris*, *Pythium ultimum*, and *Colletotrichum gloeosporioides* were among the bacteria impacted by the antifungal effect of Si/Ag-NPs. According to Jamdagni et al. (2018), ZnO-NPs also performed well against *Penicillium expansum*, *F. oxysporum*, *B. cinerea*, *A. niger*, and *A. alternata*. Furthermore, ZnO-NPs showed strong antifungal efficacy against *Aspergillus fumigates*, as demonstrated by Shinde (2015). ZnO-NPs have significant antifungal activity against *Aspergillus nidulans*, *Aspergillus flavus*, *Rhizopus stolonifera*, and *T. harzianum* (Gunalan et al., 2012). Dimkpa et al. (2013) reported that ZnO-NPs are efficient antifungally against *F. graminearum*. Furthermore, Hamza et al. (2016) found that TiO_2_-NPs have fungicidal properties against *Cercospora beticola*. On the other hand, the chemically and ecologically synthesized Ag-NPs displayed different antifungal activity (Tyagi et al., 2020).

### 4.3 Bacterial and viral infection

Bacteria are ubiquitous and can harm fungi, plants, and animals (Frey-Klett et al., 2011). In a bacterial cell, plasmids’ mobile genetic material outside the chromosome can transport crucial virulence or biological regulatory components (Partridge et al., 2018). Prophages, or bacteriophage DNA incorporated into the genome, may also be found in bacteria (Brüssow et al., 2004). Most bacteria proliferate by binary fission, which often entails the simultaneous duplication of extrachromosomal elements and chromosomal DNA (Baron, 1996). Conversely, viruses are non-cellular infectious agents limited to replicating within living cells (Koonin and Starokadomskyy, 2016). All species, including bacteria, plants, mammals, and archaea, are susceptible to viral infection (Kheyrodin et al., 2022). They can either replicate actively and control the host’s biosynthetic processes or integrate into the host’s genome and stay dormant as a provirus (Nagy and Pogany, 2012). A latent infection may arise from the suppression of transcription of the viral gene.

Most plant viruses are single-stranded and DNA-containing retroviruses and single-stranded or double-stranded RNA viruses (Shafiq et al., 2020). Ag, Cu, ZnO, and TiO_2_ are among the metal NPs whose antibacterial and antiviral qualities have been the subject of much research (Padmavathi and Anuradha, 2022). In addition to having inhibitory solid qualities, Ag-NPs have a wide range of antibacterial activities (Chen et al., 2020; Naveed et al., 2022a; Hayat et al., 2023). Ag-NPs have antibacterial activities against *Escherichia coli* and *Bacillus subtilis* (Shehzad et al., 2018), *Staphylococcus aureus*, and *Klebsiella pneumoniae* (Hussein et al., 2019; Waseem et al., 2023). Additionally, Ag-NPs prevented the growth of three dangerous food-borne bacteria: *Pseudomonas aeruginosa*, *E. coli*, and *B. subtilis* (Mohanta et al., 2017). Ag-NPs and an Ag-chitosan composite, according to Shahryari et al. (2020), suppressed the growth of *P. syringae* bacteria. According to Dang et al. (2019), Au-NPs were also demonstrated to possess bactericidal characteristics concerning *E. coli*. A range of harmful bacteria were shown to be susceptible to the bactericidal effects of distinct metal oxide NPs, such as MgO-NPs against *Ralstonia solanacearum* (Sharma et al., 2016), Cu composites against the bacterium *Xanthomonas euvesicatoria* (Fan et al., 2021), and ZnO-NPs against *E. coli* and solanacearum (Imada et al., 2016; Attar and Yapaoz, 2018).

Plants treated with ZnO-NPs both before and during the bacterial inoculation were able to stop the *Pantonea ananatis* bacterium from proliferating throughout the maize crop (Shahid et al., 2021). ZnO-NPs were also demonstrated to efficiently reduce the bacterial blight diseases that pea plants contracted from *P. syringae* and *M. incognita* (Kashyap, 2022). Furthermore, ZnO-NP additions to the soil increased rhizospheric microbial diversity, stimulated antioxidant response and plant growth in tomato plants, and reduced the occurrence of *R. solanacearum*-caused diseases (Jiang et al., 2021). ZnO-NPs derived from *Matricaria chamomilla* flower extract were found to be bactericidal against *R. solanacearum* and to reduce bacterial wilt disease in tomato plants (Khan et al., 2021b). The same ZnO-NPs derived from *Citrus medica* peel are effective against *B. subtilis*, *Streptomyces sannanesis*, *P. aeruginosa*, *A. niger*, and *Candida albicans* (Keerthana et al., 2021). Furthermore, biogenic ZnO-NPs derived from *Trichoderma reesei*, *T. harzianum*, and co-culture, as well as the *Paenibacillus polymyxa* strain Sx3, were used to inhibit the growth of *Xanthomonas oryzae* (Shobha et al., 2020). Furthermore, several studies discovered that TiO_2_-NPs inhibited sugar beet infection (cause: *P. syringae* pv. aptata), apple scab disease (cause: *Venturia inaequalis*), Fusarium wilt diseases in tomato and potato plants (cause: *F. solani*), and bacterial blight on geranium and leaf spot on poinsettia (cause: F. solani) (Hamza et al., 2016). Rice bacterial leaf blight was shown to be significantly inhibited by AgNPs, especially Al-Ag-NPs, as revealed by Tian et al. (2022).

### 4.4 Nematode infection

Nematode infestations adversely affect plant growth and vitality in the majority of crops (El-Sappah et al., 2022a). Large feeding cells are produced by the parasites’ infection of plant roots, which reduces plant nutrition and water uptake (El-Sappah et al., 2019). This can cause plants to wilt and stunt, which makes them more vulnerable to diseases and significantly lowers their yield. Of the more than 100 species of nematodes that have been discovered, root-knot nematodes (RKNs), also called *Meloidogyne* spp., are the most harmful (El-Sappah et al., 2019), with the annual global cost of RKNs being $100 billion (Khan, 2015). Hussain et al. (2016) claim that because of their fast rate of reproduction and broad host range, RKNs are challenging to regulate (Hussain et al., 2016). Some conventional means of controlling nematodes include crop rotation, chemical nematodes, cultivating resistant plant varieties, management of resistant varieties, and managing fallow land (Sivasubramaniam et al., 2020).

To manage significant plant-parasitic nematodes, nemacids are utilized despite their severe toxicity and environmental concerns (Kiriga, 2018). A multi-site mode of action has been demonstrated for NPs, making them efficient nematicides against various parasitic plant worms (Abdollahdokht et al., 2022). Comparing Au- and Ag-NPs to synthetic and hazardous nematicides, Thakur and Shirkot (2017) claim that the former have better nematicidal activity. As promising findings in treating plant diseases caused by RKNs like *M. incognita* are being obtained, the utilization of easily accessible materials for nanotechnology is increasing, according to Sharon et al. (2010). The number of deaths from *M. incognita* J2 increased after Si-NPs were applied because the SiC-NPs significantly altered the first phase of *C. elegans* larval development (Al Banna et al., 2018). According to Thounaojam et al. (2021), ZnO-NPs have antibacterial activity against nematodes, bacteria, and fungi, including *M. incognita*. TiO_2_-NPs, on the other hand, can be used to treat nematodes and viruses (Almoneafy et al., 2023). *M. incognita* in tomato plants was susceptible to the nematicidal effects of TiO_2_ (Ardakani, 2013).

## 5 Application of nanoparticles in abiotic stresses

Among the most thoroughly researched effective NPs include nanoscale crystalline powders (Fe, Co, and Cu), metal-oxide NPs (Fe_2_O_3_-NPs, TiO_2_-NPs, ZnO-NPs, SiO_2_-NPs, CuO-NPs, and CaCO_3_-NPs), fullerols, metal-based NPs (Ag-NPs and Au-NPs), and carbon nanotubes (CNTs) (Singh et al., 2021). Because of NPs’ high surface energy and high surface/volume ratio, which enhance their heightened metabolic activity and responsiveness, plants undergo a range of consequences (Juárez-Maldonado et al., 2019). NPs swiftly agitate plants through their molecular processes (Ahmad et al., 2020). Furthermore, NMs have two functions: first, they trigger OS, which triggers plants’ antioxidant defense system to guard against ROS (**Figure 2**) (González‐García et al., 2021).

### 5.1 Drought stress

As they grow and develop, plants face various environmental problems in both natural and agricultural situations (El-Sappah and Rather, 2022). Drought is one of the most detrimental environmental conditions to plant production (Seleiman et al., 2021; El-Sappah and Rather, 2022). Approximately 80%–95% of the fresh biomass in a plant is made up of water, which is crucial for several physiological functions, such as metabolism, growth, and development (Abbasi and Abbasi, 2010; Brodersen et al., 2019). Because of this, some believe that drought is the primary environmental stressor for different plants, especially in locations that are prone to drought (Anjum et al., 2011; Diatta et al., 2020), that it poses the greatest danger to future global food security, and that it has historically caused large-scale famines. Several NPs can be used to modify drought stress; research has demonstrated that silica NPs can increase plants’ resistance to drought (Chandrashekar et al., 2023). Ashkavand et al. (2015) found that silica NPs enhanced seedling development and physiological parameters in hawthorns under drought stress. Similarly, *Triticum aestivum* showed improvements in starch and gluten levels and higher growth and yield in response to dryness and wit (Li et al., 2023d). This modification has happened because TiO_2_ can promote seed germination and seedling growth (Shafea et al., 2017). During droughts, TiO_2_ also boosts biomass in plants, preserves relative water content (RWC), and promotes antioxidant enzymes (Ostadi et al., 2022). By regulating the amount of proline and promoting proline production, jute seedlings treated with hydroxyapatite NPs demonstrated enhanced drought resistance (Das et al., 2016).

Drought stress negatively affected *B. napus* while significantly impeding and delaying the growth of corn seedlings; yet, application of yttrium-doped Fe_2_O_3_-NPs improved photosynthetic apparatus, resulting in higher levels of carotenoid and chlorophyll (Palmqvist et al., 2017). By using seed reserves more quickly, ZnO in *G. max* increased seed germination percent and dry weight due to increased gibberellin activity (Sedghi et al., 2013). Similarly, Fe_2_O_3_ improved drought resistance by altering glucose metabolism and stomatal mobility (Sarraf et al., 2022). Micro ZnO has been shown in studies on maize to slow down the degradation of photosynthetic pigment, speeding up stomatal movement and photosynthesis (Chandrashekar et al., 2023). Necessary enzymes, including phosphoglucoisomerase, cytoplasmic invertase, and UDP glucose pyrophosphorylase, were modified to improve starch and sucrose production and drought stress performance (Chen et al., 2018). Zinc oxide (ZnO) might be employed as a nano agent to lessen the harmful impacts of drought stress (Jafir et al., 2024). Van Nguyen et al. (2021) report that CuO-NPs in maize favorably impact the pigment system and the ROS scavenging mechanism. It has a favorable effect on yield and aids in supplement absorption and stress resistance. Another study by Li et al. (2023b) found that foliar application of Zn-NPs, rather than ZnSO_4_, is beneficial in increasing turnip plant growth and yield under drought stress.

### 5.2 Thermal stress

From a physical standpoint, heat and cold are in the same temperature range (El-Sappah and Rather, 2022). However, in order to maintain their survival and the success of their reproduction, organisms respond to dramatically varied temperature regimes on a biological level (Acevedo-Whitehouse and Duffus, 2009). Sessile plants are subject to daily variations in temperature and seasonal variations brought on by climate change, as they cannot seek shelter (Leisner et al., 2023). According to Penfield (2008), temperature signals are a crucial factor in making decisions about the life history, including when to blossom or germinate and how long to keep seeds dormant. On the other hand, temperature cues can cause plants to begin tolerance or escape mechanisms at any stage of their life cycle (Dai Vu et al., 2019; De Smet et al., 2021; Zhu et al., 2021). Specifically, plants can acquire two tolerance mechanisms in response to almost fatal temperatures: freezing tolerance and heat stress tolerance (Hincha and Zuther, 2014; Ritonga and Chen, 2020; Haider et al., 2022). While insufficient temperature conditions might lead to optimum performance, small temperature changes within the physiological range typically result in (growth) acclimation responses (Quint et al., 2016; Casal and Balasubramanian, 2019). Hasanuzzaman et al. (2013) state that high ROS levels cause OS, which harms growth, development, and yield. As a result, plants adapt morphologically and biochemically to withstand heat stress (Bhardwaj et al., 2023).

Plants respond to this abiotic stress by triggering signaling pathways that produce osmolytes, which regulate the osmotic pressure in cells to preserve turgidity and other secondary metabolites that alter the antioxidant system (Arif et al., 2020). Increased photophosphorylation, oxygen evolution, and splitting of the water CP43 protein (Pradhan et al., 2013), nitrogen metabolism (Pradhan et al., 2014), photosynthetic capacity (Younis et al., 2020), antioxidant enzyme activity, decreased lipid peroxidation (Hassan et al., 2021a), and restoration of ultrastructural distortions of chloroplasts and the nucleus (Younis et al., 2020) are indications of the beneficial effects of NPs on plants under temperature stress. Extensive research has been conducted on the ability of TiO_2_-NPs and chitosan to withstand cold stress (Singh and Husen, 2019; Pramanik et al., 2023). Ti-NPs are helpful in cold-stressed chickpea plants for enhancing photosynthetic activity, electrolyte leakage, and membrane damage through transcriptional regulation (Singh and Husen, 2019).

The antioxidative system and transcription factors involved in the chilling response may be used by rice plants when ZnO-NPs are applied foliarly (Mirakhorli et al., 2021). Increased photosynthetic capacity in sugarcane plants under cooling stress may also result from Si-NPs (Hassan et al., 2021b). Se-NPs are effective in mitigating adverse effects, including membrane damage, reduced pollen germination, and lower crop yields, when used to strengthen the antioxidant defense system in sorghum plants subjected to high temperatures (Djanaguiraman et al., 2018). Ag-NPs encouraged morphological growth in wheat plants, protecting them against heat stress (Iqbal et al., 2019a). Zinc NPs have been shown to reduce lipid peroxidation and increase the synthesis of antioxidant enzymes, hence improving wheat’s resistance to heat stress (Hassan et al., 2018). Tomato leaves have foliar coatings of NPs that activate when temperatures rise over specific thresholds, shielding the plants from heat stress. Si-NPs could potentially be helpful in lowering heat stress (Kim et al., 2017).

### 5.3 Heavy metal stress

HM stress is one of the current negative factors affecting agricultural output (El- Sappah et al., 2021; El-Sappah et al., 2023). HM contamination has risen globally due to human activities, including industrialization and urbanization (Abbas et al., 2022a; Li et al., 2023a). Additionally, aggravating crop plant HM stress is the increasing use of chemical pesticides and fertilizers in agriculture (El-Sappah et al., 2024). HMs, such as Ag, Pb, Cd, Ni, Co, Cr, and Hg, can harm plants (Rashid et al., 2023). HMs can persist in the soil for long since they do not biodegrade (Priya et al., 2023). The mobility and availability of HMs are regulated by the rhizosphere, which has a varied root microbiome that enhances soil fertility and is heavily influenced by these substances, soil type, and biogeochemical processes (such as mineralization, precipitation, adsorption, and protonation) (Aguirre-Becerra et al., 2022). Several strategies have been put out to lessen the harmful consequences of abiotic stress (Hossain et al., 2021).

The capacity of NPs to immobilize metal ions by chemical reduction, oxidation, or absorption is another essential feature that makes them useful for soil remediation in many different nations (Rajput et al., 2022). Artificial neural patches, also known as NPs, can affect some plant processes, including the creation of apoplastic barriers that regulate the movement of water, ions, and oxygen; the regulation of metal transport genes by particular NPs that fortify the plant’s extracellular barrier against HMs; the chelation of organic acids accumulated in the cell walls of roots and leaves with HM to lessen the harm that HM stress causes to plants; and, lastly, the activation defense system. Many studies have been conducted on using NPs to reduce HM stress (Zhou et al., 2020). The HMs from the soil can be changed and absorbed by adding NPs, which lowers their mobility and bioaccumulation (Zhou et al., 2020). The amount of Cd metal available in the soil has decreased after Fe_3_O_4_-NP treatment (Wang et al., 2020). Hydroxyapatite NPs maintain soil pH and lessen the harmful effects of metals in the soil by releasing phosphate ions (Cui et al., 2018).

Furthermore, NPs stimulate the apoplast barriers to form, which reduces the concentration of HMs in the root. Additionally, plants with certain NPs that might impede HM translocation by building complexes with them can have their metal transporter genes altered to redirect HMs (Wang et al., 2021). By promoting the synthesis of organic acids, Si-NPs have lessened the harm that HM stress has produced (Cui et al., 2017; Wu et al., 2017).

### 5.4 Salt stress

An osmoregulation strategy is utilized to counteract OS brought on by the generation of ROS, which exacerbates cytotoxicity and nutritional imbalance brought on by an excessive rise in sodium (Na^+^) and chloride (Cl) (Das and Roychoudhury, 2014; Ihtisham et al., 2023). When organic molecules, including sugars, glycine betaine, amino acids, polyols, and quaternary ammonium compounds, are absorbed by a plant, their osmotic potential is reduced during the process of osmoregulation (Ghosh et al., 2021b). Another crucial tactic to counteract the ROS impact and activate the enzymatic machinery is ion homeostasis, which involves increasing the concentration of K^+^ and decreasing the concentration of Na^+^ in the cell (Tripathy and Oelmüller, 2012). By boosting osmolytes, activating specific genes, and supplying free nutrients and amino acids, NPs reduce stress (Mittal et al., 2020; Zia-ur-Rehman et al., 2023). The rate of plant transpiration, water usage efficiency (WUE), enzyme carbonic anhydrase activity, and *Cucurbita pepo*’s protective response to salt stress were all enhanced by SiO_2_-NP treatment (Siddiqui et al., 2014). Inside the ETC, TiO_2_ (anatase) blocks linolenic acid and modifies photoreduction activity (Mingyu et al., 2008).

According to a study, using a foliar spray of *Abelmoschus esculentus* ZnO enhances photosynthetic efficiency and enzymatic machinery to lessen the adverse impacts of salt stress (Alabdallah and Alzahrani, 2020). Raising photosystem II’s efficiency improved photosynthesis and positively affected plant development. In addition, it lessens membrane damage and aids in maintaining RWC (Alabdallah and Alzahrani, 2020). Similarly, foliar spraying ZnO and Si to mango seedlings improved growth circumstances by increasing carbon assimilation and nutrient absorption (Elsheery et al., 2020). Numerous studies on the application of SiO_2_ have verified the enhanced vegetative growth, increased thickness of the epicuticular wax layer, accumulation of proline, and altered expression of genes related to salt stress in *S. lycopersicum*, strawberries, and *Ocimum basilicum*, among other plants (Almutairi, 2016; Avestan et al., 2019; Oprica et al., 2021; Gou et al., 2023). According to reports, one well-known nanomaterial that may be utilized as a possible nano-agent to lessen salt stress is Ag-NPs. Ag-NPs caused *T. aestivum* to accumulate more POD, proline, and sugar, and a rise in germination (Mohamed et al., 2017).

To enhance photosynthetic carbon absorption, boost proteins and amino acids during the reproductive stage, and confer resistance to salt stress, CeO, CNTs, and graphene NPs were applied to *Gossypium* and *Catharanthus roseus* (McGehee et al., 2019; An et al., 2020). ZnO increased the germination of cumin seeds and enhanced the salt tolerance of lupine plants by lowering malondialdehyde (MDA) and Na^+^ levels. By maintaining appropriate osmoregulation, reducing MDA and Na^+^ levels, and strengthening photosynthetic system activity, n-ZnO was able to mitigate the detrimental effects of NaCl (Abdel Latef et al., 2017). According to Zulfiqar et al. (2019), NPs such as SiO_2_-NPs, Cu-NPs, Fe-NPs, Mn-NPs, C-NPs, Ti-NPs, Ce-NPs, and K-NPs were helpful in reducing the detrimental effects of salt stress in a variety of plants. El-Sharkawy et al. (2017) and Jafir et al. (2022) found that applying K-NPs topically, lowering electrolyte leakage, and raising proline and antioxidant-enzyme content, such as catalase, might all help enhance salt tolerance by foliar treatment. It has been demonstrated that cerium oxide NPs may improve mineral absorption and modify root cells in *Brassica napus*, hence boosting photosynthetic activity (Al-Khayri et al., 2023b). More and more research points to the possibility that plants might employ NPs to significantly reduce the detrimental effects of salt stress.

### 5.5 Flood stress

By modifying root cells and enhancing mineral absorption, cerium oxide NPs have been shown to help boost photosynthetic activity in *B. napus* (Al-Khayri et al., 2023b). Research suggests that treating plants with NPs may significantly reduce the detrimental effects of salt stress and modulate their responses (Zulfiqar et al., 2019). According to reports, plants stressed by flooding can gain from exposure to NPs. Ag-NPs controlled proteins, glycolysis, amino acid synthesis, and wax production in soybean plants to reduce flooding stress and promote plant development (Mustafa et al., 2015; 2016). The influence of Ag-NPs on the synthesis of the cytotoxic marker glyoxalase II 3 was one of the most noteworthy findings of proteomics. Plant tolerance to floods is increased when Ag-NPs are combined with potassium nitrate (KNO_3_) and nicotinic acid (Hashimoto et al., 2020). Soybeans’ resistance to flood stress was markedly enhanced by an additional Al_2_O_3_ metal NPs (Mustafa and Komatsu, 2016).

Additionally, because S-adenosyl-l-methionine-dependent methyltransferases and enolase are implicated, soybeans subjected to Al_2_O_3_-NPs undergo recovery (Yasmeen et al., 2016). Additionally, the use of NPs accelerates the kinetics of flooding stress recovery. NP size affects flood tolerance more than NP amount and kind, according to Mustafa and Komatsu (2016) research. When plants were exposed to flood conditions, three distinct sizes of Al_2_O_3_-NPs caused various biochemical reactions. Isobutane dehydrogenase’s catalytic activity was enhanced by applying Al_2_O_3_-NPs; however, under flood circumstances, Al_2_O_3_-NPs caused the synthesis of ribosomal proteins, and the mitochondria’s membrane permeability was increased by large concentrations of Al_2_O_3_-NPs (Mustafa and Komatsu, 2016).

### 5.6 Light stress

Plant chloroplasts use sunlight to convert carbon dioxide and water into organic matter and oxygen (Alberts, 2017). Light is an essential environmental factor for photosynthesis since it fluctuates in time and place concerning its spectral quality and light intensity (Shafiq et al., 2021). Light may be a significant abiotic stressor for plants when illumination conditions are unsuitable for their growth (Fiorucci and Fankhauser, 2017). Variable light levels can reduce photosynthetic efficiency, cause photodamage in high light, and prevent plant development in low light. When plants experience light stress, especially severe light stress, they experience photoinhibition, which results in an imbalanced energy distribution between photosystem I (PSI) and photosystem II (PSII) and a precipitous drop in photosynthetic efficiency (Walters, 2005).

Plants have developed defense mechanisms against light stress, which include the production of anthocyanins, movement of chloroplasts, generation and scavenging of chloroplastic ROS, opening and closing of stomata, and coordination of responses through systemic signaling (Shi et al., 2022). Because of their vulnerability to damage, the molecular devices that perform photosynthesis known as photosynthetic apparatuses have evolved fast responses to light stress (Shi et al., 2022). The complex states and structures of proteins linked to the thylakoid membrane are altered as one such response (Pottosin and Shabala, 2016). Plants have evolved defense strategies in response to light stress, which include the production of anthocyanins, movement of chloroplasts, stomata opening and closing, generation and scavenging of chloroplastic ROS, and coordination of responses through systemic signaling. Because of their vulnerability to harm, the molecular devices that perform photosynthesis, called photosynthetic apparatuses, have evolved rapid responses to light stress.

According to Pottosin and Shabala (2016), one such reaction is altering proteins’ complex states and structures attached to the thylakoid membrane. Increased NP concentrations may harm the photosynthetic machinery, inhibit Rubisco activity, produce toxic ROS, or interfere with CO_2_ reduction or photosynthetic electron transport (Singh et al., 2015). For these reasons, research is ongoing to determine the ideal dosages for maximizing photosynthesis (Li et al., 2023c).

Furthermore, most studies evaluate the effects of NPs on photosynthesis in stressful circumstances (like heat waves, droughts, or trace metals) or how to enhance this biological activity under regular lighting (Nayeri et al., 2023). Although the measurement’s primary focus is the energy conversion efficiency, light is not often used as a stressor in studies (e.g., high or low light intensities, harmful wavelengths, or inadequate photoperiods) (Zhang et al., 2020). Si-NPs were given to hydroponically grown wheat seedlings after exposure to UV-B light (Ruttkay-Nedecky et al., 2017; Tripathi et al., 2017).

These substances reduced the harmful effects by lowering lipid peroxidation (as MDA), raising fresh mass, leaf area, and fresh and dry leaf mass (Rizwan et al., 2019). UV-B significantly decreased total chlorophyll, and these reductions were present in Si-NPs (Tripathi et al., 2017). Additionally, fewer and milder formazan spots were observed in the leaves, suggesting a preventive function against OS caused by UV-B-induced H_2_O_2_ production. Furthermore, the loss of plant leaf thickness was compensated by less internal leaf damage from deformed palisade and mesophyll layers (Tripathi et al., 2017).

## 6 Hazard, challenges, and prospective

Because of their unique characteristics and inventive features, NPs have been widely used in various industries, including catalysts, semiconductors, environmental energy, medication delivery, and cosmetics (Nel et al., 2006). Scholars are now considering the challenges, issues, and consequences of NPs’ environmental impact because of their widespread and uncontrolled use (Gottschalk et al., 2015; Tolaymat et al., 2015). NPs improve plants’ ability to adapt to stress by altering critical metabolic pathways, boosting antioxidant defense systems, and improving processes that detoxify free radicals (Jalil and Ansari, 2019). Specific plant components such as seeds, flowers, fruits, stems, bark, leaves, peels, and roots have generated a broad spectrum of NPs, including iron, gold, platinum, palladium, silver, zinc, selenium, copper, and others (Ishak et al., 2019; Zhao et al., 2024). In stressed settings, these NPs have been demonstrated to rewire plant physio-biochemical processes (Kumari et al., 2022).

Moreover, a range of NPs have been shown to have a substantial impact on how plants react to environmental stressors such as heat stress, salinity, drought, and HM toxicity (Venkatachalam et al., 2017; Iqbal et al., 2019b; Sreelekshmi et al., 2020). Moreover, it has been shown that microorganisms, including viruses, bacteria, actinomycetes, and fungi, are all equally capable of creating NPs, which have a wide range of usefulness in easing different environmental restrictions (Ghosh et al., 2021a). Most studies involving nanomaterials in agriculture are conducted in labs or on a small scale, and these materials are still in the early phases of development (Iavicoli et al., 2017). As a result, little is known about the advantages of various NPs as well as the potential risks to the public’s health and the environment if they are used in specific applications (Kumah et al., 2023). Determining the effectiveness of these substances when added to the soil and encouraging greener practices and the sustainability of agricultural systems is thus of particular importance (Gupta et al., 2023). It is typically possible that metallic NPs have incredibly beneficial impacts on germination (Santás-Miguel et al., 2023). Regretfully, most tests are carried out under carefully monitored circumstances in laboratories. Owing to their small size, NPs present several environmental risks, such as ease of transport and dispersion, the potential to be ecotoxic, persistent in the environment, and capable of bioconcentrating or bioaccumulating in higher organisms (Ray et al., 2009). There is also a chance that these processes may be reversible.

Additional studies are needed to determine the toxicity and ecotoxicity of various NPs on land and aquatic creatures in the food chain because of these potential environmental hazards. Therefore, research on risk assessment is necessary to understand the possible impacts on non-target species and their significance for agricultural productivity and healthy soils (Chauhan and Gill, 2023). More investigation is essential to comprehend how NPs interact with cellular components and, therefore, their function in biochemical reactions through OS pathways, as they can operate more quickly than larger-sized NPs. The public’s health is in danger from ingesting contaminated food and drink, skin contact, inhalation, or exposure to NPs through polluted air (Handy and Shaw, 2007). Thus far, genotoxicity, pulmonary illnesses, OS, and lipid peroxidation have been reported as consequences.

DNA mutations have been reported to cause cell harm in other, more significant cases (Bhabra et al., 2009). Therefore, one must take caution while using NPs until the safety parameters required for proper usage are established. After the environmental and public health effects are understood, nations, accountable institutions, or groups can create the necessary laws and regulations for using these NPs in agriculture.

## 7 Conclusion

Numerous environmental influences on plants limit and reduce agricultural crop yields. Plants are exposed to two environmental stressors: biotic and abiotic stress. Nanotechnology is one of the many approaches to tackle these pressures. In the face of a burgeoning global population, agricultural nanotechnology has become a powerful tool for aiding crops and increasing agricultural output. It also helps plants adapt to environmental conditions. The molecular effects of NPs on diverse plant species are not the same. For example, certain plants are more vulnerable to the ROS mode generation by NPs, while others undergo metabolic changes and differential expression of defense-related genes. The usage of NPs provides benefits; however, there are also downsides for public and plant health. Plants are vulnerable to various molecular effects caused by NPs, which begin with the activation of the ROS mode, progress to metabolic changes, and conclude in the differential expression of specific defense genes. Despite their many applications, NPs are detrimental to human and plant health.
